# The Patient’s Conceptions of Wound Treatment with Negative Pressure Wound Therapy

**DOI:** 10.3390/healthcare2030272

**Published:** 2014-07-21

**Authors:** Ann-Mari Fagerdahl

**Affiliations:** Department of Surgery, Department of Clinical Science and Education, Södersjukhuset, Karolinska Institutet, SE-171 77 Stockholm, Sweden; E-Mail: ann-mari.fagerdahl@ki.se; Tel.: +46-70089-1063; Fax: +46-8616-2460

**Keywords:** negative pressure wound therapy, NPWT, wound treatment, patient’s conceptions

## Abstract

During the last two decades, additional methods have been developed in wound care where traditional treatments have been insufficient. Negative pressure wound therapy (NPWT) is one such method. This method has been described in multiple studies, but still, many pieces of the puzzle are missing to get a complete picture of NPWT’s impact on the patient’s health-related quality of life and how the patient experiences the treatment. The purpose of this study was to describe the patient’s conceptions of wound treatment with NPWT. The study was inspired by phenomenography, and eight interviews were conducted with patients treated with NPWT. The results of the study were grouped into two main categories: stress and adaptation. Three descriptive categories were presented under stress: personal environment, competence of the nursing staff and organization and continuity of the dressing changes. Two descriptive categories were presented under adaptation: knowledge and creativity and confidence with the healthcare. Patients were affected by the treatment, and at times, the stress meant that they had difficulty coping. The most common source of stress observed in this study was the care environment, particularly the organization of the dressing changes and deficiencies in the healthcare personnel’s competence.

## 1. Introduction

Throughout history, wounds and, particularly, slow-healing wounds have been a cause of suffering and great distress to unfortunate victims. During the last two decades, additional methods have been developed in areas where traditional wound treatment has been insufficient. Negative pressure wound therapy (NPWT) is one such method. This method has been explored in multiple studies, and it has been shown that NPWT may be stressful to the patient. However, there are still many pieces of the puzzle missing to get a complete picture of the impact on the patient’s health-related quality of life (HRQoL) and how the patient experiences this treatment.

The NPWT method consists of a device that creates a vacuum in the wound using a wound filler of polyurethane foam, polyvinyl alcohol foam dressing or gauze. The foam, or gauze, is adapted exactly after the size of the wound, and then, the wound filler and the entire wound are covered with a transparent adhesive drape. A hole is cut in the drape and a suction tube adapted. The tube is connected to the vacuum machine, and a subatmospheric pressure is applied.

NPWT has been in clinical use for wound management since 1995, and the first scientific documentation originates from 1997 with the work of Argenta and Morykwas [1]. Since then, thousands of articles have been published, but only a small fraction of the literature focuses on the patient’s conceptions and experiences of the treatment.

The impact on the HRQoL during NPWT has been explored qualitatively in only a few studies. Abbotts showed that the treatment with NPWT was experienced as stressful, especially regarding the impact on daily life and the organization of dressing changes [2]. An interview study by Bolas and Holloway confirms these findings, but also emphasizes the technical aspect of NPWT and describes the feelings of distress associated with its use [3].

Upton, Stephens and Andrew described that the NPWT system can cause patients to feel anxious, due to both the patient and the health professional being unfamiliar with this form of treatment. Furthermore, they described that the treatment can also restrict the patient’s daily care and wider social life, which may result in a negative self-image and low self-esteem. They also emphasize the need for more knowledge, particularly exploring the patient’s experience throughout the treatment process in order to minimize the negative effects of NPWT [4].

The World Union of Wound Healing Societies (WUWHS) consensus document on NPWT states that NPWT can have a positive impact on a patient’s HRQoL [5]. However, Ousey, Cook and Milne conclude in their review of the impact of NPWT on the patient’s HRQoL that it is not possible to determine whether the impact is positive, neutral or negative based on existing research [6]. Since the amount of research focusing on the patient’s experiences and that the existing literature presents varying results with both negative and positive impact on the patient’s HRQoL, it is necessary to conduct more qualitative research on the effects of NPWT. The aim of this study was to describe the patient’s conceptions of wound treatment with NPWT.

## 2. Methods

In this study, a phenomenographic approach was used. Phenomenography is a research method that explores the qualitatively different ways in which people perceive a specific phenomenon. Fundamental in phenomenography is to find the variation of people’s conceptions of this phenomenon [7]. In this study, the phenomenon is wound treatment with NPWT.

### 2.1. Participants

The participants were purposefully selected to ensure variation with respect to gender, age, wound type, type of NPWT device and treatment time, in accordance with the phenomenographic methodology [7,8]. Nineteen patients treated with NPWT during 2006 were asked to participate in the study, and in total, eight patients agreed (Table 1).

**Table 1:** Demographic and medical data of the participants (*n* = 8). NPWT, negative pressure wound therapy.

Variables		Number
Gender	Men	6
	Women	2
Age	Range	20–73
	Median	66
Wound type	Post-operative wound infection	2
	Diabetic foot ulcer	1
	Pressure ulcer	1
	Traumatic wound	2
	Open abdomen	2
NPWT pump type	Portable	4
	Stationary	4
Treatment time (days)	Range	2–42
	Median	17

The NPWT system used was, in four cases, a portable vacuum-assisted closure (VAC) device (ActiV.A.C., KCI Inc, San Antonio, TX, USA) and in four cases, a larger stationary pump (InfoV.A.C., KCI Inc, San Antonio, TX, USA). The dressings were changed twice weekly. The dressing changes were performed as an inpatient treatment for patients with the stationary pumps and at the outpatient clinic for patients with portable machines. The healthcare personnel performing the wound treatment were physicians of different specialties, registered nurses and nurse’s aides at a large emergency city hospital. The hospital had no formal requirement that the personnel should have received specialized education in wound care, so knowledge and competence varied and was dependent on the individual’s experience and own interest.

### 2.2. Data Collection

Interviews were conducted in the period of June–November, 2006. A non-structured interview procedure was used, developing new questions following earlier answers, until no further information was received. All interviews began with one open question, where the participants were asked to talk freely about their conceptions of NPWT in general. The interview was expanded by follow-up questions regarding the injury, the wound healing process and the experience of being treated with NPWT. Six of the interviews were conducted at the hospital and two were telephone interviews. All interviews were conducted by the same researcher and lasted from seven to 43 min. The interviews were tape-recorded and transcribed verbatim.

Initially, six interviews were conducted and analyzed. Then, two more additional interviews were conducted, and after analysis, no new data was received, indicating a satisfying saturation of the material [9].

### 2.3. Data Analysis

Data analysis was conducted according to the phenomenographic method [8]. In all phases of the analysis, discussions took place between the researcher and co-workers, until consensus was reached.

The transcribed interviews were initially read several times to get familiar with the content and to obtain a sense of the whole. When a deeper understanding of the content was reached, distinct statements of conception were compared. Statements with similar content were grouped together and categorized into five labelled descriptive categories. These categories were thoroughly examined and discussed to ensure that they were distinctly separated from each other. In the next phase, the underlying meaning on an abstract level of the descriptive categories was analyzed, discussed and formulated into two main categories. Finally, the whole material was analyzed again to confirm the correlation between the statements of conception, descriptive categories and the main categories with the original text of the transcribed interviews.

### 2.4. Ethical Considerations

All participants were given written and verbal information, and their informed consent was obtained. Confidentiality was assured by decoding the interviews and all research data were kept in locked cabinets. Ethical approval was obtained by the local Ethics Committee (2006/571-31/2).

## 3. Results

The findings in this study show that being treated with NPWT was perceived by the participants as stressful, and at times, the stress meant that they had difficulty coping. The ability to adapt to the prevailing circumstances had a major effect on their conceptions and experiences of the wound treatment process. The descriptive categories presented in the result comprise the participants’ conceptions as identified in their responses (Table 2).

**Table 2:** Patients’ conceptions of being treated with NPWT: main categories and description categories.

Description Category	Main Category
Personal environment	Stress
Competence of the nursing staff	
Organization and continuity of the dressing changes	
Knowledge and creativity	Adaptation
Confidence with the healthcare	

### 3.1. Stress

The majority of the participants perceived treatment with NPWT as being stressful, but worth the inconvenience.

#### 3.1.1. Personal Environment

The participants’ personal environment was affected by the treatment in physical, mental, social and spiritual aspects. The participants particularly perceived physical discomfort during the treatment. Some of the participants described the treatment as being painful, especially during the dressing changes, but the majority did not perceive the treatment as painful at all. One participant even expressed himself so well that the staff was surprised by the fact that he did not have any pain:

“...about the abdomen...I don’t feel that I...I never had any pain...in the abdomen...all doctors asked but do you not have any pain there...?”

The most frequently described problem when being treated with NPWT was the inconvenience of being attached to a machine all the time. This was particularly disturbing to the participants treated with the larger stationary pump. The participants with the smaller portable pump, however, described an inconvenience when carrying it for a longer time, even when it felt light at first. The machine also affected some issues of daily life, like getting dressed and undressed and taking a shower. One man described frustration in the prolonged time required for performing everyday tasks:

“Most difficult this period was taking a shower…with a plastic bag…or thinking that the tube enters somewhere…and there will leak in water…if it gets soaked it must be replaced. So I put on two socks and then a plastic bag…oh, it was the greatest project…and what I have missed most of all…is not to sleep but to stand on two naked feet in the shower…”

#### 3.1.2. Competence of the Nursing Staff

The participant perceived the competence and knowledge of the treatment as being rather varying and that there were major differences within the personnel who fully mastered the treatment compared to those that did not. Several participants described this as feeling like guinea pigs:

“It is not so many that feels...you know of the staff that knows this inside out yet, so they are experimenting a bit”

“…the staff…they said they did not know much…so they were also curious to know more about the machine…”

The competence of the staff was perceived by the majority of the participants as being inadequate, and they described this as very troublesome. The participants, however, also described being tolerant and understanding regarding the deficiencies in the competence of the staff, since they were aware of that the treatment was new, some even expressed an interest in being part of the staff’s education.

“…it was a bit…fascinating. Yes, there were several people in the OR and they were invited to watch the dressing changes…on some occasions there was a flow of visitors asking if they could take a look…well, it can be fun with a little public but finally only four persons at a time were allowed to watch the dressing changes as it became crowded I suppose…”

#### 3.1.3. Organization and Continuity of the Dressing Changes

All of the participants described the continuity of the dressing changes as troublesome, particularly since there were so many people involved in their care and no one with the full responsibility.

The participants who had their dressing changes performed in the operation room (OR) ward described the waiting as most stressful in the process of dressing changes. The procedure was planned in the so-called emergency list at the OR and prioritized together with all other emergency cases in need of surgery. All of the participants experienced being given lower priority to have to wait for a long time for each dressing change. They all expressed this not being a great problem when being treated once or twice, but for longer treatment periods with many dressing changes, it became a major concern. Particularly problematic was when being forced to fast all day and the dressing change was postponed until the next day:

So that a…well…that part was an inconvenience, to have to wait not knowing if the change of dressing could be done that day…all of a sudden it could not be done and then you did not know when next a change could be performed…well you must get a scheduled time for the change of dressing.

### 3.2. Adaptation

Despite the stressful impact the treatment had on the participants, the majority perceived the treatment as being positive and that they were able to adapt and to manage the stress.

#### 3.2.1. Knowledge and Creativity

Several of the participants described the importance of knowledge, both the knowledge within the staff, but also their own knowledge of the NPWT technique and their understanding of their own wound treatment. The participants received information regarding the treatment several times, however of varied content, and it is difficult to understand. The healthcare personnel who was informing also showed clear shortcomings in knowledge. One participant perceived that the staff was taking much for granted and did not understand that the patients had difficulties comprehending the information. Furthermore, the reduced health condition that several of the participants had was considered a reason for the perceived lack of information given and the understanding of that information.

The participants talked about several problems with daily living during treatment, but also how they, in a creative way, went about to solve these problems. They showed great creativity when trying to adapt to the situation and make everyday life as manageable as possible, both on their own, but also together with the healthcare personnel. Some participants treated with the larger stationary pump had different ways of making the pump more mobile:

“...then I went and experimented a bit on the ward so it resulted in that we took this vacuum pump and put on one of those IV-poles and then it went after all...it was...I was able to walk around and it up and...”

“…but I learnt to put the bed there (closer to the shower room, authors’ comments). I put the wire under the door so I could take a shower on my own.”

#### 3.2.2. Confidence with the Healthcare

The participants said that, from the very beginning, they had had great confidence in the treatment and in the healthcare staff, and when the wound started to heal, they felt faith in the future. One participant had had the wound for a long time and was willing to try just about anything to see an improvement. Particularly, participants treated with open abdominal wounds described the treatment and trust in healthcare as giving them hope for recovery. One participant said that he, before treatment with NPWT, had been lying with an open abdomen and experienced how the intestines virtually fell out when he moved. With NPWT, he got the feeling that his body was whole again and with that, the agony he felt disappeared and the hope of recovery was lit:

No, it was that feeling…those first days…that everything leaks out of you…it was literally speaking only the peritoneum which held the intestines in place, and it leaked and smelled…you felt this is not going to work…almost a sort of deadly anxiety, I must say. I thought I wouldn’t survive…despite everyone saying to the contrary…When they applied this VAC dressing it felt more like it was a part of my body, somehow…The body felt whole again. This increased my well-being psychologically…from thinking “This is the end” to suddenly feeling “This is not so bad”.

## 4. Discussion

The results of this study show that the participants treated with NPWT perceived the treatment as positive and effective, despite stress in the form of physical strain and the inconvenience of being connected to the unit around the clock. These strains were managed by the participants’ feeling of the fundamental belief in the treatment and healthcare and that they had trust that their wounds would heal. Moreover, they perceived knowledge of the treatment method as important and contributing to their ability to creatively solve problems that arose during the treatment. The participants perceived the inadequate and varied skills of the healthcare staff and the organization and continuity of the dressing changes as being the most troublesome aspect.

### 4.1. Stress

The participants stated that treatment with NPWT was stressful to them, which is in accordance with other research focusing on patients’ experiences of traditional wound treatment [10,11,12].

The most troublesome for the participants during treatment was the organization of the dressing changes, particularly when performed in the OR ward. This problem has also been described by Abbott and by Bolas and Holloway [2,3]. It is important to facilitate the care of these patients and to minimize stress. By planning the dressing changes as elective operations in the surgical planning schedule, the risk of being postponed can be reduced. This could give the patients a better ability for themselves to prepare for the dressing change, which could result in a greater sense of control.

Another issue contributing to stress during treatment was the inadequate and varying competence of the healthcare personnel. This is also a well-described problem with NPWT treatment in the literature [3,13]. It is a major concern when apparently insufficiently-educated personnel handle advanced treatment, such as NPWT. Unfortunately, problems with the staff’s lack of skills are not unique to NPWT, but also occur in other wound treatment methods, as confirmed by previous research [14,15]. Graham [16] pinpoints the importance of sufficient education before applying the therapy, especially since incorrect use could seriously harm the patient. Graham suggests an educational program according to the theories by Patricia Benner [17] with different knowledge levels, from novice to expert. According to the ethical principle of non-maleficence, embodied by the phrase “first, do no harm”, it is essential for healthcare to ensure that the personnel has adequate knowledge of the equipment and method used, to avoid the risk of harming the patient.

The participants’ description of pain during treatment and, particularly, the absence of pain are worth mentioning. Procedural pain during dressing changes when treated with NPWT was earlier described in the literature [4]; however, studies of pain during the entire wound treatment process have shown varying results, and some studies even indicate that NPWT as a treatment may, in fact, ease the wound pain rather than enhance it [4,13].

### 4.2. Adaptation

The participants had to adapt to the current situation to manage the stress involved in the treatment to maintain a balance and the conception of health. The first step towards adaptation for the patients was receiving sufficient knowledge and information regarding the treatment. Edward, Moffat and Franks point out the importance of adequate information for the experience and management of the strain that wound treatment may have on the patient [18]. They also emphasize the varying quality of information provided. In their study, only one fifth of the patients had received some form of written information. The participants in this study expressed that poor general status of health during treatment was one explanation of difficulties to comprehend received information. Having the possibility of written information in addition to verbal could facilitate the patients’ understanding and allow them to process the information in a longer time span.

When feeling confident in managing the treatment, the participants became inventive and creative in dealing with different obstacles that arose in everyday life. Knowledge and confidence were key factors for managing and coping in a positive way with stressful issues during treatment, which is in concordance with other studies of NPWT [4,13] and in wound management, in general [11,12,19].

### 4.3. Methodological Considerations

Why is it important to know the patient’s conceptions of NPWT? There is an old saying: “The cure is worse than the disease”. This means that the treatment itself can be effective, but at the same time, so incredibly stressful to the individual patient that it is just not worth it. It is only the patient who is an expert of his/her own body and own conceptions, and therefore, research must be based on a patient’s perspective. Thus, using phenomenography as a research method and purposive sampling is appropriate, particularly since it is possible to identify a variation of conceptions, which is the main objective of phenomenography as a research method [7,8]. To ensure clinical credibility, the whole process of analysis was performed in close collaboration with co-workers and other wound experts, and the process has been thoroughly described in the Methods section.

Regarding the transferability of the result, it should only be seen as an awareness-raising of the knowledge of wound patients and not as a representative experience of all patients treated with NPWT. However, since the participants in this study were selected with a large variation concerning age, gender, different wound types, different treatment times and different types of NPWT machines, the result describes a wide range of conceptions, which may be transferred to patients treated with NPWT in other settings.

One limitation of qualitative research may be that it is the interviewer who is the main instrument in the acquisition of knowledge. It is important that the researcher is aware of his/her role in order to obtain scientific knowledge, also adhering to ethical considerations, during the research interview. By recording and transcribing the interviews verbatim, the credibility of collected material may be enhanced. To ensure a sufficient amount of material, two additional interviews were performed. These interviews did not change the findings, so that the feeling of saturation of the material was achieved [9]. Another limitation of this study may be the rather short interviews, often due to the poor health status among several of the participants. However, the objective with this study was not to perform in-depth interviews, only to describe a variation of conceptions among patients treated with NPWT.

## 5. Conclusions and Relevance for Practice

The findings in this study show that patients were negatively affected by treatment with NPWT and, at times, the stress meant that they had difficulty coping. The largest source of stress observed in this study was the clinical setting, particularly the organization of the dressing changes and deficiencies in healthcare personnel’s competence.

These findings have relevance for the practice by demonstrating the importance of the organization of the treatment, especially the dressing changes, and highlight the insufficient knowledge and skills in wound management of the healthcare personnel that must be addressed.
